# Data on chemical compositions and fermentation quality of silages made from low-market-value vegetables supplemented with potato protein concentrate, a byproduct of starch production

**DOI:** 10.1016/j.dib.2018.11.043

**Published:** 2018-11-12

**Authors:** Michiko Okubo, Kazunari Sato, Shunsuke Matsuda, Takayoshi Masuko, Kousaku Souma

**Affiliations:** Department of Northern Biosphere Agriculture, Faculty of Bioindustry, Tokyo University of Agriculture, 196 Yasaka, Abashiri 099-2493, Japan

## Abstract

This data article reports the chemical compositions (protein, fat, fiber, ash, lactic acid, acetic acid, propionic acid, butyric acid and valeric acid) and fermentation quality, represented by V-value determined from the proportion of ammonia nitrogen in total nitrogen and volatile fatty acid contents, in silages prepared from low-market-value vegetables (carrot roots, cabbage leaves, and radish leaves). Potato protein concentrate, a byproduct of starch production from potato tuber, was used to supplement the protein contents in the silages. The first type of silage was produced by fermentation of a mixture of wheat bran and either carrot, cabbage, or radish without supplemental potato protein. The second type of silage was produced by fermentation of a mixture of wheat bran and either carrot, cabbage, or radish with supplemental potato protein. The third type of silage was produced by mixing the first silage type with unfermented potato protein. Chemical compositions and fermentation quality of the three silage types are provided in table formats.

**Specifications table**

**Table:** 

Subject area	*Agricultural science*
More specific subject area	*Livestock science*
Type of data	*Tables*
How data were acquired	*General chemical compositions of silages were measured using methods published by the Association of Official Analytical Chemists**[1]**. pH value of the silage was measured using a pH meter. Lactic acid content was measured using the colorimetric method of Barker and Summerson**[2]**. Volatile fatty acids were measured using gas chromatography (GC-12A; Shimadzu Co., Ltd. Kyoto, Japan)**[3]**. Volatile basic nitrogen was measured using steam distillation method**[5]*.
Data format	*Analyzed*
Experimental factors	*Silages were produced by fermentation of a mixture of wheat bran and vegetables and/or potato protein in plastic silos.*
Experimental features	*Chemical compositions and fermentation quality of silages.*
Data source location	*Abashiri, Hokkaido, Japan*
Data accessibility	*All data are presented in this article.*

**Value of the data**

• The data represent chemical composition of silages produced from low-market-value vegetables and support further studies on estimating their nutritional effect on livestock production.
• The data are preliminary records on the effect of potato protein concentrate, which is an additive used as a protein content supplement, in the silage production.
• The data can be used to develop a recipe for silage production by using waste from agricultural vegetable production.

## 1 Data

This data article contains tables showing chemical compositions (Table 1) and fermentation qualities (Table 2) of silages produced by fermentation of a mixture of wheat bran and low-market-value vegetables and/or potato proteins, a byproduct of potato starch production.

**Table 1:** Chemical composition of pre-ensiled vegetables and silages.

	Dry matter	Crude protein	Crude fat	Crude fiber	ADF	NDF	NFE	Crude ash
	(% FM)	(% DM)	(% DM)	(% DM)	(% DM)	(% DM)	(% DM)	(% DM)
Pre-ensiled								
Carrot	15.7	7.3	7.3	9.0	16.7	19.4	51.7	8.9
Cabbage	17.1	18.9	1.9	14.2	19.6	20.1	36.1	11.7
Radish	8.0	20.3	3.4	13.5	28.2	27.7	23.9	30.9
Wheat bran	6.0	17.6	5.5	11.0	11.1	44.1	53.3	6.6
PPC	5.7	86.7	5.1	1.5	9.0	43.5	0.0	4.4
Type 1 (w/o PPC) a								
Carrot	26.3	14.5	4.4	3.7	15.7	33.3	63.5	6.2
Cabbage	23.9	18.0	5.0	2.6	15.9	36.9	61.5	6.7
Radish	24.2	19.2	6.0	1.6	17.9	33.2	57.2	10.4
Type 2 (w/ PPC) b								
Carrot	20.6	18.0	4.2	1.9	15.0	37.5	61.8	5.9
Cabbage	20.4	21.9	4.6	2.7	15.2	34.8	58.0	6.5
Radish	20.7	23.1	5.8	2.2	19.4	35.9	50.8	11.7
Type 3 (type 1+PPC) c								
Carrot	26.4	18.1	4.5	3.6	15.4	33.8	60.1	6.1
Cabbage	24.1	21.4	5.0	2.5	15.5	37.2	58.3	6.6
Radish	24.4	22.6	5.9	1.6	17.4	33.7	54.2	10.1

FM, fresh matter; DM, dry matter; ADF, acid detergent fiber; NDF, neutral detergent fibers; PPC, potato protein concentrate.

a Silages made by mixture of each vegetable and wheat bran.

b Silages made by mixture of each vegetable and wheat bran and PPC.

c Mixed feeds of type 1 silage and PPC without fermentation.

**Table 2:** Fermentation quality of type 1 and type 2 silages.

	Type 1 (w/o PPC)			Type 2 (w/ PPC)		
	Carrot	Cabbage	Radish	Carrot	Cabbage	Radish
pH	3.96	4.05	3.92	3.93	3.89	4.15
Lactic acid (% DM)	6.51	6.61	6.50	6.66	6.18	7.18
Acetic acid (% DM)	2.61	2.11	2.06	2.75	1.90	2.70
Propionic acid (% DM)	0.18	0.07	0.02	0.08	0.12	0.15
Butyric acid (% DM)	0.09	0.05	0.07	0.14	0.15	0.00
Valeric acid (% DM)	0.10	0.05	0.04	0.06	0.09	0.04
Volatile basic nitrogen (% Total nitrogen)	6.3	9.2	8.2	6.6	8.1	8.3
V-score	87.6	86.8	90.2	90.7	88.3	89.7

DM, dry matter; PPC, potato protein concentrate.

## 2 Experimental design, materials, and methods

### 2.1 Experimental design

In this study, imperfect vegetables with low market values were used to produce silages. Potato protein concentrate, which is a byproduct of potato starch production, was added to the silages to enhance the protein contents in silage products.

### 2.2 Materials

Low-market-value vegetables were employed to prepare the silages. Carrot roots and cabbage leaves were obtained from the Fruit and Vegetables Center of JA Shari (Shari, Hokkaido, Japan), radish leaves were obtained from Tanji Farm (Abashiri, Hokkaido, Japan), and potato protein concentrate was provided by the Starch Factory of JA Shari (Shari).

### 2.3 Silage preparation

In this experiment, we prepared three types of silage. The first type was prepared by fermentation of a mixture (80:20 w/w) of either carrot roots, cabbage leaves, or radish leaves and wheat bran. The second type was prepared by fermentation of a mixture (80:15:5 w/w/w) of either carrot roots, cabbage leaves, or radish leaves, wheat bran, and potato protein concentrate. The third type was prepared by mixing (95:5 w/w) the first type of silage and potato protein concentrate.

Silage was ensiled on 29 September 2012. The vegetables were cut into pieces approximately 2–3 cm long before ensiling in 220 L FRP silos. Each silage was prepared in five silos. The filling weight was 125–130 kg of fresh matter. The silos were fermented for 120 days.

### 2.4 Chemical analyses

The pre-ensiled vegetables and silages were air dried at 60 °C for 48 h to prepare air dry samples. General composition and acid and neutral detergent fibers were measured in the pre-ensiled vegetables and silages. In silages, pH, lactic acid, volatile fatty acids, and volatile basic nitrogen levels were measured and the V-score was determined using extracts obtained by homogenizing 50 g fresh samples in 100 mL of distilled water.

Dry matter content was determined by measuring the weight before and after the drying process at 135 °C for 2 h [1]. Crude protein, crude fat, crude fiber, and ash were measured as described by AOAC [1]. Neutral detergent fiber and acid detergent fiber contents were analyzed using the procedures described by van Soest et al. [4]. The pH value was determined using a pH meter (HM-25G; DKK-ToA Corporation, Tokyo, Japan). Lactic acid content was determined using the colorimetric method of Baker and Summerson [2]. Other volatile fatty acids were measured using gas chromatography (GC-12A; Shimadzu Co., Ltd. Kyoto, Japan) [3]. Volatile basic nitrogen content was measured using the steam dilution method [5]. V-score, which was used to access the silage fermentation quality, was determined from the proportion of volatile basic nitrogen in total nitrogen and volatile fatty acid contents in the silage [5].
